# Retraction Note: Small Diameter Blood Vessels Bioengineered From Human Adipose-derived Stem Cells

**DOI:** 10.1038/s41598-022-10006-4

**Published:** 2022-04-05

**Authors:** Renpeng Zhou, Lei Zhu, Shibo Fu, Yunliang Qian, Danru Wang, Chen Wang

**Affiliations:** 1 Department of Plastic and Reconstructive Surgery, Shanghai 9Th People’s Hospital, Shanghai Jiao Tong University School of Medicine, 639 Zhi Zao Ju Road, Shanghai, 200011 People’s Republic of China; 2 Department of Plastic & Cosmetic Surgery & Burn, The Third Affiliated Hospital of SunYat-Sen University, 600 Tian He Road, Guangzhou, 510000 People’s Republic of China

Retraction of: *Scientific Reports* https://doi.org/10.1038/srep35422, published online 14 October 2016

The Editors have retracted this Article.

Concerns were raised that some of the data in this Article, in particular those in Figures 2B, 5B and 5C, have been published previously [1, 2]. Additionally, the hUASMCs/calponin image in Figure 2B appears to correspond to a different sample than the same image in [1]. The Editors therefore no longer have confidence in the results and conclusions presented.

Renpeng Zhou, Lei Zhu, Danru Wang and Chen Wang agreed with the retraction and its wording. Shibo Fu and Yunliang Qian did not respond to the correspondence from the Editors about this retraction.
